# The cross-cultural adaptation and psychometric evaluation of the Chinese version of the stigma scale for caring for individuals with sexually transmitted infections (STISS)—student version: a translation and validation study

**DOI:** 10.3389/fpsyg.2026.1858646

**Published:** 2026-07-21

**Authors:** Jiahao Shi, Youbei Lin, Chuang Li, Yinghui Gong, Hongyu Li, Yue Pan

**Affiliations:** 1 The First Affiliated Hospital of Jinzhou Medical University, Jinzhou, China; 2 Jinzhou Medical University, Jinzhou, China; 3 Department of Social Psychology, Nankai University, Tianjin, China

**Keywords:** nursing student, psychometrics, public health, quality of care, sexually transmitted infection

## Abstract

**Background:**

Stigmatization of patients with sexually transmitted infections remains prominent, whereas previous measurement tools have mostly focused on single-disease populations and lack a systematic assessment of the broader population of individuals with sexually transmitted infections.

**Objective:**

To translate the Stigma Scale for Caring for Individuals with Sexually Transmitted Infections (STISS) – Student Version and evaluate its reliability and validity among nursing students in China.

**Methods:**

This study recruited 413 nursing students through convenience sampling based on Kendall’s sample size estimation method. The original version of the scale was translated according to the Brislin guidelines, and content validity and the feasibility of the translation were validated through expert consultation. Structural validity was assessed using exploratory factor analysis and confirmatory factor analysis. Reliability was tested using internal consistency reliability.

**Results:**

The Chinese version of the STISS scale demonstrated good psychometric properties. The Cronbach’s alpha coefficient was 0.921. The four-factor exploratory factor model explained 58.947% of the total variance, indicating a robust factor structure. Confirmatory factor analysis showed the following fit indices: *χ*^2^/df = 1.078; RMSEA = 0.019; CFI = 0.994; TLI = 0.993; GFI = 0.927; AGFI = 0.907. All indices were within acceptable ranges, and both convergent validity and discriminant validity were adequately confirmed.

**Conclusion:**

This study strictly followed the Brislin translation model and successfully introduced the STISS scale, which demonstrated strong reliability and validity within the Chinese cultural context. It is highly suitable for measuring the level of stigmatization of individuals with sexually transmitted infections among nursing students in China.

## 1 Introduction

Sexually transmitted infections (STIs) refer to diseases primarily transmitted through sexual contact (Van Gerwen et al., 2022). STIs are caused by over 30 different microorganisms, including bacteria, viruses, and parasites (Aggarwal et al., 2022; Kungu et al., 2026). The global burden of STIs is mainly attributed to eight pathogens: four curable bacterial and parasitic infections (syphilis, gonorrhea, chlamydia, trichomoniasis) and four incurable viral pathogens—hepatitis B, herpes simplex virus (HSV), human immunodeficiency virus (HIV), and human papillomavirus (HPV) (Kungu et al., 2026; Isara and Baldeh, 2021). The transmission modes of STIs can be broadly categorized into three types (Elendu et al., 2024):

(1) Bacterial STIs: Syphilis, caused by *Treponema pallidum*, is a typical example of a bacterial STI. Primary syphilis presents as a painless genital ulcer or chancre, while secondary syphilis is characterized by systemic disease with rashes, fever, and mucocutaneous lesions.

(2) Viral STIs: This includes HSV, HPV, and HIV, with HPV being the most common viral STI globally. It encompasses various genotypes, some of which are carcinogenic and can lead to cervical cancer, anal cancer, and oropharyngeal cancer. HPV infections can also cause genital warts, cervical dysplasia, or asymptomatic infections, depending on the virus genotype and host immune response (Aral et al., 2015).

(3) Parasitic STIs: Although less common than bacterial and viral STIs, they still present significant health risks and control challenges. Trichomoniasis is caused by the protozoan parasite Trichomonas vaginalis, leading to vaginal discharge, itching, and discomfort in women, while men may experience urethritis (Baird et al., 2012). Additionally, STIs can be transmitted through sexual contact, pregnancy, blood transfusions, or contact with contaminated blood products (Açıl and Üstgörül, 2026). STIs can lead to numerous severe health problems (Elendu et al., 2024), such as pelvic inflammatory disease (PID), cancer, infertility, ectopic pregnancy, stillbirth, and neonatal death.

According to the World Health Organization (WHO) report (Van Gerwen et al., 2022), it is estimated that approximately 1 million people worldwide are infected with sexually transmitted diseases every day. Influenced by unique cultural, social, economic, and epidemiological factors, the situation of sexually transmitted diseases varies significantly across different regions of the world (Elendu et al., 2024). For example, high-income countries in North America, Western Europe, and parts of the Asia-Pacific region have relatively effective control of sexually transmitted diseases (STD) due to strong healthcare infrastructure, comprehensive sexual health education programs, and widespread screening, diagnosis, and treatment services (Alam et al., 2010). In contrast, low- and middle-income countries, especially those in sub-Saharan Africa, South Asia, and Latin America, face great challenges in controlling sexually transmitted diseases (STD) due to factors such as poverty (limited healthcare resources and insufficient funding for STD control programs), shortages of healthcare personnel, barriers to accessing preventive services, gender inequality, and stigma (Martin et al., 2021). In addition to regional differences and resource limitations, stigma and discrimination are also among the major challenges in the global prevention and control of sexually transmitted diseases (Elendu et al., 2024). The term “stigma” originates from Greek, referring to a physical mark. In ancient Greece, marks were branded or carved onto the bodies of slaves, criminals, and traitors to indicate their disgraceful and dishonorable status (Fan, 2023). In 1963, Goffman first proposed the concept of “stigma” and introduced it into the field of psychology, defining it as a process in which the majority in society label certain groups or individuals as untrustworthy and undesirable, often resulting in damaged reputation, loss of status, discrimination, and exclusion (Zhang, 2021). With the advancement of research, Goffman and other scholars have revised, refined, reinterpreted, and redefined the concept of stigma from different perspectives (Ruan, 2025; Zhang, 2024). Goffman (1967) proposed a new perspective, suggesting that the existence of “stigma” is not merely a problem of the stigmatized individuals, but is largely caused by deficiencies in social rules and public order (Guan, 2007). Jones (1984) argued that stigma links individuals with undesirable characteristics (i.e., stereotypes). Stafford and Scott (1986) suggested that stigma arises from the conflict between individual attributes and social norms. Crocker et al. (1998) defined stigma as a social identity in which individuals (or groups), due to characteristics that differentiate them from others (or other groups) in a specific context, are rejected and lose part of their value (Corrigan, 2004). Corrigan et al. (2005) categorized stigma from a social cognitive perspective into two types: public stigma (also known as social stigma) and self-stigma. Public stigma refers to the negative stereotypes held by out-groups toward stigmatized individuals; self-stigma refers to the stigmatizing attitudes and behaviors that stigmatized individuals hold toward themselves or their group after internalizing public stigma, including perceived prejudice, stereotypes, and discrimination from others and society, as well as internalized outcomes such as shame, blame, hopelessness, guilt, and fear, and negative self-cognitions such as self-devaluation and low self-efficacy (Corrigan et al., 2005).

Experiences of stigma may adversely affect the quality of life and mental health of patients with sexually transmitted infections, and hinder their access to and adherence to healthcare services. Existing studies have provided supporting evidence for this. A study by Elendu et al. (2024), Nigerian scholars, showed that stigma and shame related to sexually transmitted diseases affect social isolation, interpersonal difficulties, and psychological distress, thereby increasing the burden on patients with sexually transmitted infections. A study by Mohammadi et al. (2026), Iranian scholars, on women with HPV infection found that after diagnosis, patients often experienced negative emotions such as helplessness, confusion, impaired interpersonal relationships, and decreased self-esteem, accompanied by a significant perception of social stigma, and often developed insecurity and distrust toward their partners. The impact of stigma leads them to tend to provide inconsistent information during medical consultations to conceal their disease status, thereby weakening the effectiveness of doctor–patient communication and further resulting in adverse outcomes such as delayed healthcare seeking or even avoidance of medical care.

Additionally, Nyblade et al. (2019), American scholars, pointed out in their study that stigma in healthcare institutions is particularly severe, and common driving factors include negative attitudes, fear, beliefs, lack of awareness of the disease itself and stigma, insufficient clinical management capacity, as well as institutionalized procedures or practices. These are specifically manifested as refusal to provide healthcare services, provision of low-quality care, and physical and verbal abuse. A study by Turan et al. (2017), American scholars, indicated that anticipated and experienced stigma from healthcare workers may lead to a series of adverse outcomes among people living with HIV (PLWH), including lack of trust in physicians, reduced quality of life, and poor adherence to antiretroviral therapy (ART). One of the reasons for this phenomenon is that, during clinical care, healthcare workers may attempt to protect themselves from contamination and may inadvertently label individuals with sexually transmitted infections in a stigmatizing manner (Açıl and Üstgörül, 2026). In addition, some healthcare providers may also experience moral distress due to moral judgments about disease-related behaviors, thereby contributing to stigma, affecting their professional function as effective caregivers, and ultimately weakening the quality of care. Therefore, stigmatizing attitudes among healthcare workers are not only an important factor influencing patient health outcomes but also a key entry point for developing interventions, and scientifically measuring them is of significant importance.

Although existing studies have revealed the adverse effects of stigma on individuals with sexually transmitted infections from multiple perspectives, stigma, as a complex and multidimensional psychosocial construct, is highly subjective and context-dependent, and its measurement and assessment still face certain challenges. At present, there is still a lack of measurement tools for assessing healthcare workers’ stigmatizing attitudes and behaviors toward STI patients. In China, several scales on stigma toward individuals with sexually transmitted infections have been developed or introduced; however, these instruments have limitations such as small sample sizes during development, non-adherence to methodological guidelines, and lack of a theoretical framework. In addition, most of these scales focus on specific populations such as people living with HIV, and scales targeting the overall STI population remain relatively scarce. Therefore, it is important to develop or introduce a measurement tool that can assess healthcare workers’ stigmatizing attitudes and behaviors toward the overall population of individuals with sexually transmitted infections.

In 2026, the research team led by Açıl and Üstgörül (2026) successfully developed the Sexually Transmitted Infections Stigma Scale (STISS) to assess healthcare workers’ perceptions of stigma and discrimination related to sexually transmitted infections. This 20-item scale consists of four dimensions: “Blame,” “Concealment,” “Personal Response,” and “Segregation from Society.” Compared with existing scales, the STISS not only focuses on healthcare workers’ stigmatizing attitudes and behaviors toward patients with HIV/AIDS but also includes their attitudes and behaviors toward individuals with all sexually transmitted infections. The scale has undergone rigorous psychometric validation and demonstrated strong reliability and validity. Therefore, this study aims to translate the STISS into Chinese and evaluate its reliability and validity among nursing students in China. Through this study, we aim to provide strong support for assessing stigma-related tendencies toward sexually transmitted infections, help undergraduate nursing students enhance their sensitivity to sexually transmitted diseases, and educate them to adopt an equal and non-discriminatory attitude in healthcare services, thereby further improving the quality of nursing care.

## 2 Methods

### 2.1 Design

This study consisted of two main stages: cross-cultural adaptation and psychometric evaluation, with the latter conducted using a cross-sectional design.

### 2.2 Participants and sample

From March to April 2026, a cross-sectional study was conducted using a convenience sampling method to select third- and fourth-year nursing students from four medical universities in Northeast China. The sample consisted only of nursing students (as they were easily accessible to the researchers), while medical students or other allied health students were not included, as they represent the primary pre-professional group with clinical exposure to infectious diseases. The inclusion criteria were as follows: full-time enrolled nursing students; prior clinical internship or clinical placement experience; and voluntary participation in the study. The exclusion criteria were as follows: students currently participating in other research projects; students on leave; and students with psychiatric disorders (such as mood disorders and depression).

The sample size was estimated using Kendall’s method, which recommends that the sample size should be 5–10 times the number of questionnaire items (May and Looney, 2022). Based on preliminary calculations and an anticipated 20% attrition rate, the scale used in this study contained 20 items, resulting in a required sample size range of 120–240 participants. In addition, to meet the minimum sample size requirements for exploratory factor analysis (≥100 participants) and confirmatory factor analysis (≥200 participants) (Flora and Flake, 2017), a total of 413 nursing students were ultimately recruited.

### 2.3 Measurement instruments

(1) General Information Questionnaire: Developed by the research team, this questionnaire includes items such as gender, age, place of origin, and grade level.

(2) Chinese version of the STISS (C-STISS) (Açıl and Üstgörül, 2026): The scale consists of four dimensions and a total of 20 items, as follows: Blame (Items 1–9); Concealment (Items 10–12); Personal Response (Items 13–17); Segregation from Society (Items 18–20). The five-point Likert scale is used, where: Strongly disagree = 1 point; Disagree = 2 points; Neutral = 3 points; Agree = 4 points; Strongly agree = 5 points. The total score ranges from 20 to 100. A higher score indicates a greater tendency of healthcare workers to stigmatize individuals with sexually transmitted infections.

### 2.4 Translation and cross-cultural adaptation of the STISS

**Figure 1:**
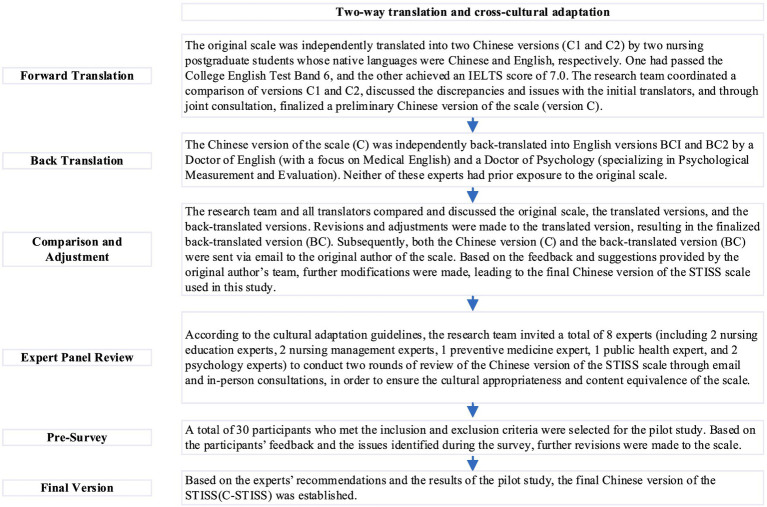
The translation and cross-cultural adaptation process of the STISS scale.

Our research team contacted the scale authors via email to obtain the scale and its usage instructions (note: the original scale development study had been approved by the local institutional review board). After authorization was obtained, the scale was translated using the “forward–backward translation” method of the Brislin model (Flora and Flake, 2017), a classic procedure in cross-cultural research used to ensure semantic and conceptual equivalence of scales, as shown in Figure 1. According to cultural adaptation guidelines (Beaton et al., 2000), the research team invited a total of eight experts (including two nursing education experts, two nursing management experts, one preventive medicine expert, one public health expert, and two psychology experts) to conduct two rounds of review of the Chinese version of the STISS scale via email and in-person consultation, in order to ensure the cultural appropriateness and content equivalence of the scale. Information about the experts is presented in Table 1.

**Table 1:** Expert basic information.

Expert ID	Age	Gender	Years of work experience	Educational background	Professional title	Research area
A	55	Female	37	Master’s degree	Professor	Preventive Medicine
B	37	Female	16	Master’s degree	Lecturer	Nursing Education
C	55	Female	36	Master’s degree	Associate Professor	Nursing Management
D	45	Male	15	PhD	Lecturer	Health Social Psychology
E	52	Male	25	PhD	Associate Professor	Public Health
F	45	Female	20	PhD	Associate Professor	Nursing Education
G	45	Female	22	PhD	Professor	Nursing Management
H	60	Female	35	Master’s degree	Lecturer	Clinical Psychology

### 2.5 Formal survey and quality control

First, the research team contacted the relevant school administrators and obtained research permission. Subsequently, all investigators were uniformly trained, including standardized procedures and precautions for participant recruitment, informed consent procedures, explanation of the study, and data collection. The questionnaire was designed using the Chinese online survey platform “Questionnaire Star” and a QR code was generated for data collection. Before completing the questionnaire, participants were presented with an electronic informed consent form on the first page of the survey. The consent form clearly stated the study purpose, procedures involved, potential risks and benefits, data confidentiality assurances, voluntary participation, and the right to withdraw at any time without penalty. Participants were required to read the information and indicate their willingness by selecting the option “I agree to participate.” Only those who provided affirmative electronic consent were allowed to proceed to the survey items. All questionnaires were completed anonymously and used solely for scientific research purposes. A total of 454 questionnaires were distributed in this study. After excluding questionnaires completed in less than 2 min and invalid responses, 413 valid questionnaires were collected. The effective response rate was 90.97%.

### 2.6 Data analysis

#### 2.6.1 Research tools

Statistical analyses were performed using SPSS 31.0 and AMOS 29.0. Skewness and kurtosis of each item were calculated, and commonly used statistical criteria (absolute values of skewness and kurtosis within ±2) were applied to assess whether the data approximately conformed to a normal distribution (Li et al., 2025). Categorical variables were presented as counts and percentages. Continuous variables that followed a normal distribution were expressed as mean ± standard deviation; those that did not follow a normal distribution were reported as median and interquartile range. A *p*-value < 0.05 was considered statistically significant.

#### 2.6.2 CTT

This study used Classical Test Theory (CTT) to analyze the scale data, assessing reliability through internal consistency reliability. The Delphi method was used to evaluate the content validity of the scale. Structural validity was tested through Exploratory Factor Analysis (EFA) and Confirmatory Factor Analysis (CFA), and further evaluations were conducted on the scale’s discriminant validity and convergent validity. Item analysis was performed using high-low group comparisons and correlation analysis.

The application of this scale in previous studies remains limited, and its factor structure has not been sufficiently validated in terms of stability and generalizability. In addition, this study involved cross-cultural adaptation and revision of the scale within the Chinese cultural context, during which linguistic and contextual modifications to the items may have affected their underlying structure. Therefore, it cannot be assumed *a priori* that the original factor structure would remain applicable in the present study setting. Accordingly, exploratory factor analysis (EFA) was conducted to re-examine the underlying factor structure of the revised scale, rather than directly adopting the original dimensional framework.

### 2.7 Ethical statement

This study was approved by the Ethics Review Committee of Jinzhou Medical University (JZMULL2025294) and adhered to its ethical guidelines. Informed consent was obtained from all participants to ensure confidentiality and anonymity. The research was conducted in accordance with the ethical principles outlined in the Declaration of Helsinki.

## 3 Results

### 3.1 Demographic characteristics

A total of 413 nursing students were included in this study, with an average age of 20.95 years. Of the participants, 92.0% were female, and 78.9% of the students came from urban areas. The details are shown in Table 2.

**Table 2:** General characteristics of participants (*n* = 413).

Variable	Category	*N*(%)/mean ± SD
Age (year)		20.95 ± 0.906
Gender	Male	33(8%)
	Female	380(92%)
Home residence	Rural	87(21.1%)
	Urban	326(78.9%)
Grade	Third	182(44.1%)
	Fourth	231(55.9%)

### 3.2 Cross-cultural adaptation

Firstly, for Item 1 (“I am more confident in caring for a person with a physical illness rather than a person with STIs”), based on expert feedback and internal discussions within the research team, the term “Physical illness” was considered too vague. It was translated as “bodily diseases,” which was too broad, and “physiological diseases,” which was too narrow. Neither accurately reflected the original intent. Therefore, to ensure clarity and comprehensibility of the comparison, the team decided to use “other patients” to avoid ambiguity and emphasize the distinction from individuals with sexually transmitted infections (STIs). Secondly, for Items 2 (“I may unwillingly exhibit negative body language when I have to care for an individual with STIs”) and 3 (“Despite my professional roles, I may react negatively toward individuals with STIs”), based on expert feedback and internal discussions, the words “negative” and “negatively” in the original items, which could be translated as “negative” or “adverse,” were found to be inappropriate in the Chinese context. Therefore, following expert suggestions, the research team used the term “aversive” in the translation to more neutrally and objectively reflect the unconscious psychological rejection and physical expressions that respondents might exhibit when caring for individuals with STIs. Regarding the term “professional roles” in Item 3, it was considered slightly unclear, so upon expert advice, we replaced it with “healthcare professionals” to ensure clearer expression. Finally, for Item 5 (“I have difficulty in showing understanding to the individual with STI”), the research team felt that “showing understanding” typically refers to both cognitive and emotional understanding and response to someone’s situation, which includes cognitive understanding of the situation and some degree of emotional resonance. Based on expert feedback, the team adjusted the translation to “acceptance” to better reflect an individual’s tendency to either accept or reject STI patients on a value or attitude level. All these modifications were approved by the original scale’s author and have been supported in the subsequent data analysis.

### 3.3 Item analysis

Before item analysis, normality tests were conducted on all 20 items. The results showed that both skewness (−0.955 to −0.509) and kurtosis (−0.511 to 0.514) were within the absolute value range of ±2, indicating that the data were approximately normally distributed (Li et al., 2025), as detailed in Supplementary material 1. The collected scale responses were ranked in descending order based on total scores, with the top 27% defined as the high-score group and the bottom 27% as the low-score group. Since all items approximately followed a normal distribution, independent-samples *t*-tests were used for analysis. The results showed that all items exhibited significant differences between the two groups (*p* < 0.001), indicating good discriminant validity. Details are shown in Table 3.

**Table 3:** Item analysis results of the Chinese version of the stigma scale for caring for individuals with sexually transmitted infections (C-STISS).

Item	High group *M* ± SD	Low group *M* ± SD	*t*	df	*p*	Mean diff.	95% CI of diff.	
							Lower limit	Upper limit
B1	4.55 ± 0.500	2.87 ± 1.109	−14.720	157.982	*p* < 0.001	−1.681	−1.907	−1.456
B2	4.65 ± 0.480	3.07 ± 1.134	−13.655	152.986	*p* < 0.001	−1.578	−1.807	−1.350
B3	4.53 ± 0.501	2.80 ± 1.107	−15.199	158.422	*p* < 0.001	−1.733	−1.959	−1.508
B4	4.59 ± 0.493	2.79 ± 1.109	−15.846	156.926	*p* < 0.001	−1.805	−2.030	−1.580
B5	4.62 ± 0.487	2.82 ± 1.243	−14.350	147.711	*p* < 0.001	−1.797	−2.045	−1.550
B6	4.58 ± 0.496	2.89 ± 1.011	−15.984	165.446	*p* < 0.001	−1.691	−1.899	−1.482
B7	4.59 ± 0.493	2.96 ± 1.189	−13.487	151.577	*p* < 0.001	−1.630	−1.868	−1.391
B8	4.57 ± 0.498	2.93 ± 1.239	−13.073	149.343	*p* < 0.001	−1.638	−1.885	−1.390
B9	4.60 ± 0.491	3.00 ± 1.105	−14.126	156.902	*p* < 0.001	−1.604	−1.828	−1.379
C1	4.46 ± 0.685	2.67 ± 1.187	−13.918	181.594	*p* < 0.001	−1.793	−2.047	−1.539
C2	4.38 ± 0.787	2.59 ± 1.218	−13.133	194.020	*p* < 0.001	−1.791	−2.060	−1.522
C3	4.16 ± 0.826	2.64 ± 1.213	−11.024	199.750	*p* < 0.001	−1.522	−1.794	−1.250
PR1	4.34 ± 0.757	2.57 ± 1.152	−13.673	195.846	*p* < 0.001	−1.772	−2.028	−1.517
PR2	4.12 ± 0.795	2.54 ± 1.220	−11.488	194.896	*p* < 0.001	−1.573	−1.843	−1.303
PR3	4.32 ± 0.751	2.36 ± 1.153	−15.116	194.833	*p* < 0.001	−1.956	−2.211	−1.701
PR4	4.37 ± 0.750	2.47 ± 1.138	−14.788	196.149	*p* < 0.001	−1.896	−2.148	−1.643
PR5	4.24 ± 0.855	2.41 ± 1.166	−13.457	207.331	*p* < 0.001	−1.831	−2.099	−1.563
SS1	4.37 ± 0.673	2.71 ± 1.165	−13.116	181.803	*p* < 0.001	−1.659	−1.908	−1.409
SS2	4.41 ± 0.755	2.70 ± 1.212	−12.693	190.105	*p* < 0.001	−1.704	−1.968	−1.439
SS3	4.39 ± 0.728	2.71 ± 1.188	−12.802	188.264	*p* < 0.001	−1.677	−1.935	−1.418

B, blame; C, concealment; PR, personal response; SS, segregation from society.

Subsequently, a corrected item-total correlation (CITC) test was conducted, as shown in Supplementary material 1. The results indicated that the CITC values for all items were above the 0.30 threshold. Items such as B4 (“When caring for individuals with STIs, I try to avoid communicating with them”) (*r* = 0.708) and B3 (“Despite being a healthcare professional, I may react aversively toward individuals with STIs”) (*r* = 0.676) showed strong correlations with the total score. Furthermore, no significant increase in Cronbach’s *α* coefficient was observed after deleting any item, so all items were retained. In summary, the results from both the extreme group comparison and item-total correlation analysis indicated that all 20 items had good discriminant ability and internal consistency, and thus all items were retained for further analysis.

### 3.4 Reliability analysis

The overall Cronbach’s α coefficient for the Chinese version of the STISS was 0.921. The reliability coefficients for each dimension were as follows: 0.902 for “Blame,” 0.794 for “Concealment,” 0.858 for “Personal Response,” and 0.771 for “Segregation from Society.” All reliability indicators were higher than the reference standard of 0.7 (Liu et al., 2024), indicating that the scale has good internal consistency.

### 3.5 Validity analysis

In this study, eight experts were invited to evaluate the cultural appropriateness and relevance of each item on the C-STISS using a four-point rating scale (1 to 4 corresponding to “Not relevant” to “Very relevant”). The Scale-level Content Validity Index (S-CVI) was 0.913, and the Item-level Content Validity Index (I-CVI) ranged from 0.875 to 1.00, all exceeding the acceptable thresholds (S-CVI ≥ 0.90; I-CVI ≥ 0.78).

### 3.6 Exploratory factor analysis

**Figure 2:**
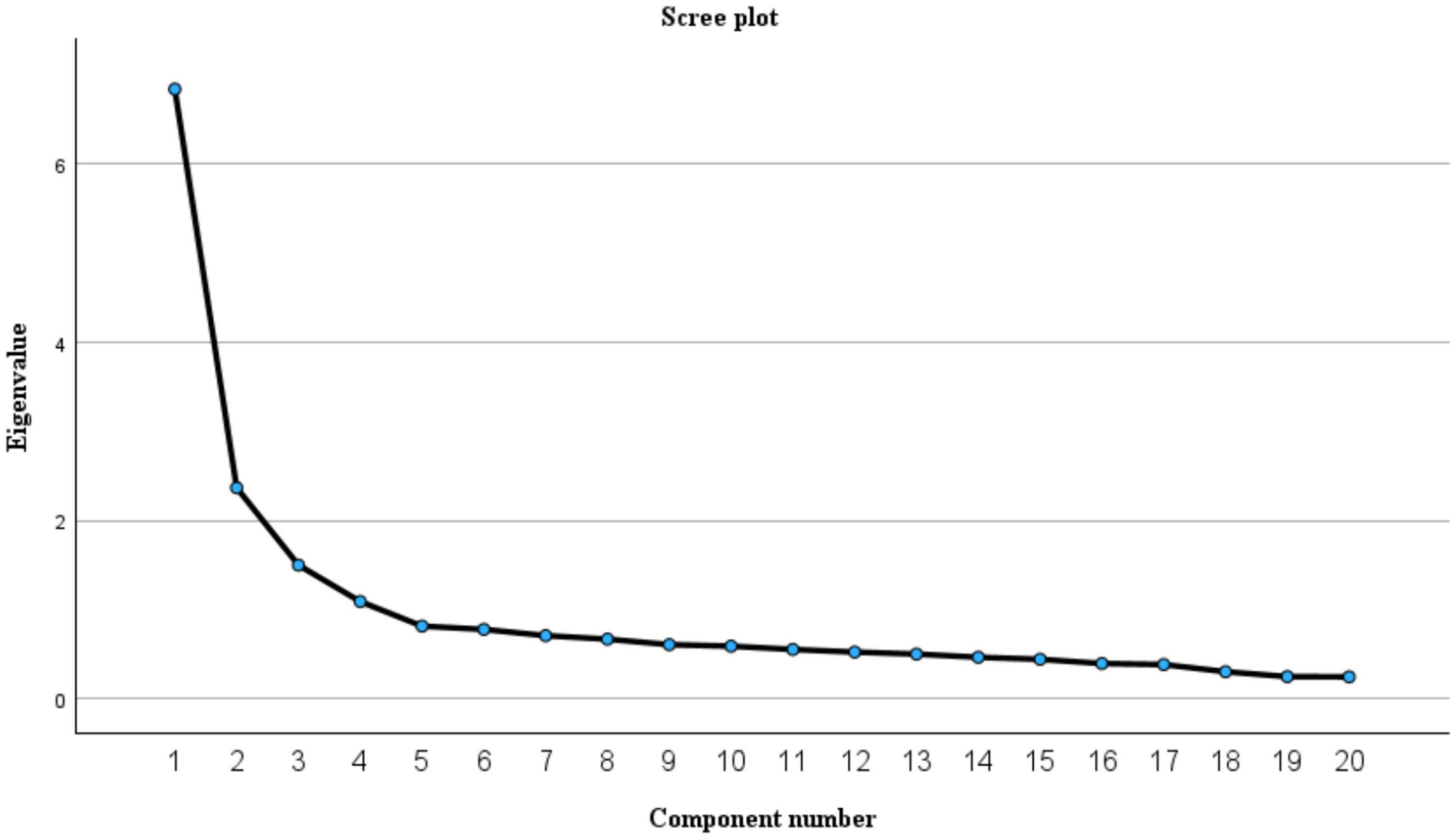
Scree plot of the four-factor C-STISS (*n* = 206).

The total sample (*N* = 413) was randomly divided into two subsamples at a 1:1 ratio: 206 cases were used for exploratory factor analysis (EFA), and 207 cases were used for confirmatory factor analysis (CFA). Before conducting exploratory factor analysis (EFA), the Kaiser–Meyer–Olkin (KMO) measure of sampling adequacy and Bartlett’s test of sphericity were performed. A KMO value greater than 0.7 and *p* < 0.05 are generally considered acceptable for factor analysis (Gebremedhin et al., 2022). The results showed that the KMO value was 0.895, and Bartlett’s test of sphericity yielded *χ*^2^ = 1655.581 (*p* < 0.001). Using principal component analysis and varimax rotation, four factors with eigenvalues greater than 1 were extracted, explaining a cumulative variance of 58.947%. The assignment of dimensions and items was consistent with the original scale. All factor loadings exceeded 0.5, and no cross-loadings were observed (Wang et al., 2014), as detailed in Figure 2 and Table 4.

**Table 4:** Factor loadings of the C-STISS from exploratory factor analysis (*n* = 206).

Item	Factor 1	Factor 2	Factor 3	Factor 4
B1	0.702			
B2	0.781			
B3	0.789			
B4	0.778			
B5	0.750			
B6	0.769			
B7	0.755			
B8	0.724			
B9	0.669			
C1			0.712	
C2			0.782	
C3			0.814	
PR1		0.726		
PR2		0.692		
PR3		0.606		
PR4		0.605		
PR5		0.647		
SS1				0.700
SS2				0.748
SS3				0.667

### 3.7 Confirmatory factor analysis

**Figure 3:**
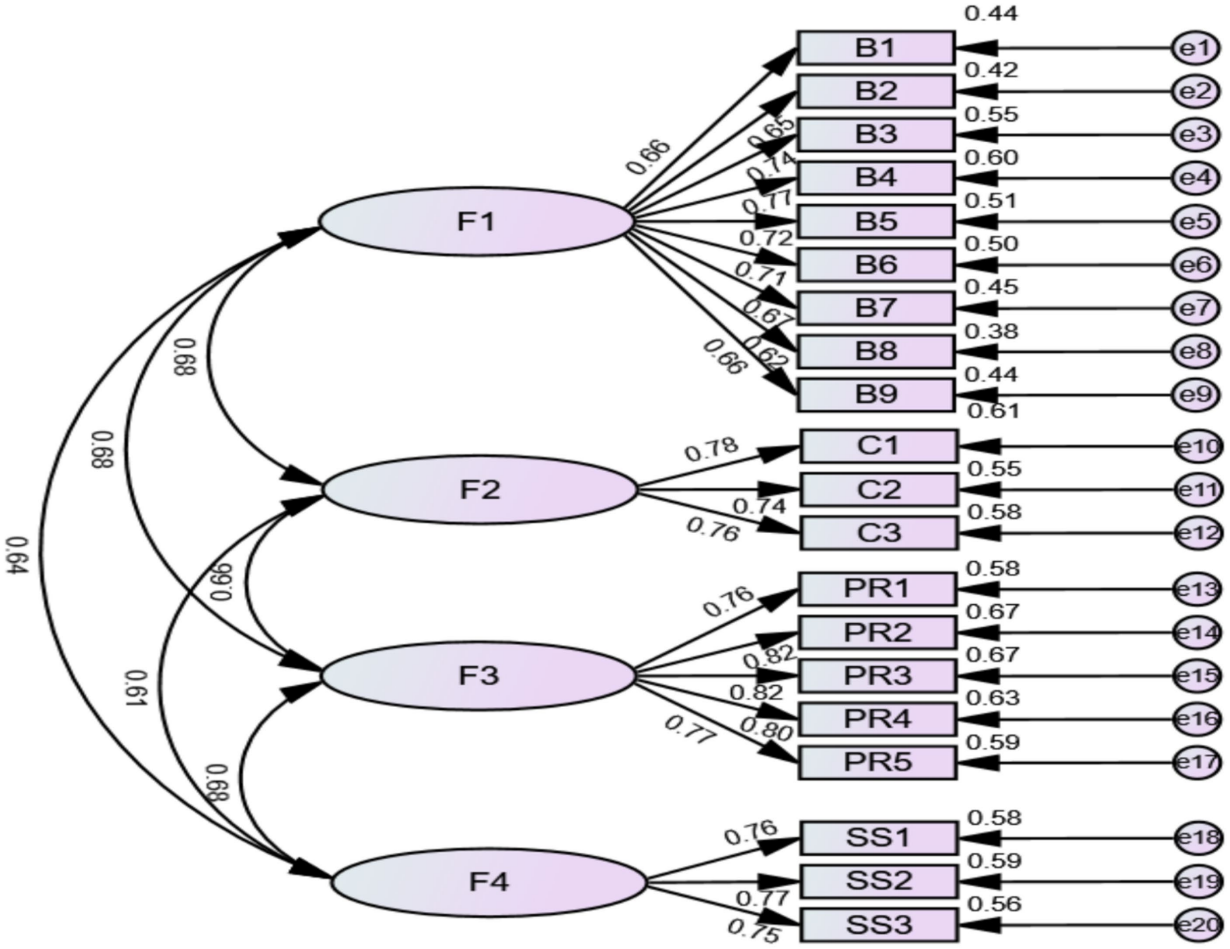
Standardized factor loading diagram of the four-factor C-STISS (*n* = 207). F1: Blame; F2: Concealment; F3: Personal Response; F4: Segregation from Society.

Confirmatory factor analysis (CFA) was used to test the item-factor structure of the STISS scale. The model fit indices were as follows: *χ*^2^(207) = 176.789; *χ*^2^/df = 1.078; RMSEA = 0.019; CFI = 0.994; TLI = 0.993; GFI = 0.927; AGFI = 0.907, all greater than 0.9. These results indicate a good model fit, as shown in Figure 3.

### 3.8 Convergent validity and discriminant validity

Based on the standardized factor loadings from CFA, the Composite Reliability (CR) values ranged from ≥0.7 (0.803–0.894). The Average Variance Extracted (AVE) values ranged from >0.4 (0.537–0.627), indicating that the C-STISS demonstrates good convergent validity, as shown in Table 5. Additionally, the square root of the AVE for each dimension was greater than the corresponding correlation coefficients between dimensions, as detailed in Table 6.

**Table 5:** Convergent validity of the Chinese STISS scale.

Items	Path	Factors	Std. estimate	S. E.	*P*	CR	AVE
B1	←	F1	0.663			0.88	0.537
B2	←	F1	0.645	0.118	***		
B3	←	F1	0.74	0.128	***		
B4	←	F1	0.773	0.135	***		
B5	←	F1	0.715	0.134	***		
B6	←	F1	0.707	0.113	***		
B7	←	F1	0.673	0.124	***		
B8	←	F1	0.62	0.123	***		
B9	←	F1	0.661	0.117	***		
C1	←	F2	0.781			0.804	0.578
C2	←	F2	0.742	0.096	***		
C3	←	F2	0.759	0.091	***		
PR1	←	F3	0.76			0.894	0.627
PR2	←	F3	0.816	0.09	***		
PR3	←	F3	0.82	0.093	***		
PR4	←	F3	0.795	0.089	***		
PR5	←	F3	0.768	0.093	***		
SS1	←	F4	0.759			0.803	0.575
SS2	←	F4	0.766	0.109	***		
SS3	←	F4	0.75	0.108	***		

*** indicates *p* < 0.001.

**Table 6:** Discriminant validity of the Chinese STISS scale.

Factor	F4	F3	F2	F1
F4	0.759			
F3	0.679***	0.792		
F2	0.614***	0.655***	0.759	
F1	0.636***	0.683***	0.677***	0.733

*** indicates *P* < 0.001; F1: Blame; F2: Concealment; F3: Personal Response; F4: Segregation from Society.

## 4 Discussion

### 4.1 The C-STISS items demonstrate good discrimination, reliability, and validity

This study evaluated the discriminant validity of the scale using the critical ratio (CR) method and item–total correlation analysis. Independent-samples t-tests were used to compare differences between the high-score and low-score groups. The results of the CR method showed statistically significant differences between the two groups for all items (all *p* < 0.001), indicating strong discriminative power of the items (Liu et al., 2024). The corrected item–total correlation (CITC) values ranged from 0.531 to 0.667 (all > 0.3), meeting the criterion for item retention and indicating good homogeneity between each item and the overall scale (Tong et al., 2021). Compared with the original scale (CITC: 0.441–0.760), the CITC range of the Chinese version was slightly more concentrated. This difference may be attributed to improved consistency in item comprehension resulting from semantic adjustments during the cross-cultural adaptation process.

Content validity was assessed to verify whether the scale items accurately reflected the construct being measured. In this study, eight experts were invited to evaluate the content validity of the scale and to provide cultural adaptation suggestions. The results showed that the S-CVI of the translated scale was 0.913, and the I-CVI values ranged from 0.875 to 1.00, indicating good overall content validity (Rollo et al., 2025). These findings suggest that the C-STISS is highly accepted by professionals, is linguistically and culturally appropriate, conforms to Chinese language norms, and is easy to understand, effectively reflecting nursing students’ stigma toward individuals with sexually transmitted infections.

Structural validity is a theoretical form of validity that reflects the conceptual framework of the construct under investigation. In this study, exploratory factor analysis (EFA) with varimax rotation was used to explore the underlying structure, and four latent factors were identified: Blame, Concealment, Personal Response, and Segregation from Society. These factors were highly consistent with the corresponding dimensions of the original English version of the scale. In addition, all rotated factor loadings exceeded 0.5, and no cross-loadings were observed, fully meeting psychometric standards. The KMO value was 0.895, and Bartlett’s test of sphericity was statistically significant, indicating that the data were suitable for factor analysis. The four-factor model explained 58.947% of the total variance, reflecting the multidimensional nature of nursing students’ stigma toward individuals with sexually transmitted infections. The relatively high cumulative variance indicates strong structural validity of the Chinese version of the scale. Compared with the original scale (68.8%), the cumulative explained variance of the Chinese version was slightly lower but still higher than the commonly accepted minimum threshold in psychometrics (40%) (Mvududu and Sink, 2013), suggesting overall stability of the factor structure. Such differences are common in cross-cultural scale adaptation studies and may be related to variations in the interpretation and response patterns of stigma-related concepts across cultural contexts. For example, Chinese nursing students may interpret dimensions such as “Blame” or “Concealment” in a more situational manner, leading to a more dispersed factor loading pattern for certain items and slightly reduced explanatory power. Confirmatory factor analysis (CFA) was used to examine whether the relationship between factors and their corresponding indicators fit the theoretical framework of the study. The CFA results showed that RMSEA was below 0.08, and GFI, TLI, AGFI, and CFI were all above 0.9 (Li et al., 2025; Asadizaker et al., 2023). All indices reached the recommended thresholds, indicating a satisfactory model fit and confirming that the scale has strong structural validity.

Convergent validity assesses whether items measuring the same latent construct are appropriately grouped. In the C-STISS, the CR values for all four factors were above 0.6, and the AVE values were all greater than 0.5 (Tong et al., 2021), indicating good convergent validity. In addition, the Fornell–Larcker criterion showed that the square root of AVE for each construct was greater than the correlation coefficients between that construct and other latent variables, confirming that the C-STISS can effectively discriminate between different latent variables (Liu et al., 2024). In summary, the results of this study confirm the validity and applicability of the C-STISS as a tool for assessing the level of stigma toward individuals with sexually transmitted infections among Chinese nursing students. The findings suggest that the scale can accurately reflect nursing students’ stigma toward individuals with sexually transmitted infections and demonstrates satisfactory reliability and validity.

### 4.2 The application value and significance of the C-STISS

The cross-culturally adapted and validated C-STISS developed in this study provides a reference measurement tool for assessing the level of stigma toward individuals with sexually transmitted infections among Chinese nursing students. In current clinical practice, stigmatizing attitudes among healthcare professionals toward patients with sexually transmitted infections may affect their nursing behaviors, communication quality, and patients’ healthcare experiences; therefore, objective assessment of such stigma is of great importance (Açıl and Üstgörül, 2026). In nursing education settings, this scale can help educators identify differences in students’ attitudes across various dimensions (e.g., blame attribution, avoidance, or concealment tendencies), thereby providing a basis for targeted curriculum design and intervention strategies. By quantifying stigma levels, educators can implement more focused anti-stigma training and improve nursing students’ humanistic care abilities and professional competencies.

In addition, from a cross-cultural perspective, the C-STISS not only achieves linguistic translation of the original scale into the Chinese context but also validates the stability and variation of the stigma structure related to sexually transmitted infections across different cultural backgrounds during the adaptation process. In the Chinese cultural context, stigma related to sexually transmitted infections is often closely associated with moral judgment, social responsibility attribution, and feelings of shame, which may strengthen tendencies toward blame and concealment. Therefore, during the translation and revision process, expressions that could cause ambiguity or cultural inappropriateness were modified to ensure that the items retained their original theoretical meaning while aligning with the cognitive patterns of Chinese nursing students. The development of this tool provides a methodological foundation for future cross-cultural comparative studies and contributes to a deeper understanding of the role of cultural factors in the formation and expression of stigma.

### 4.3 Limitations

Although this study has several limitations, they should be acknowledged. First, the cross-sectional design and convenience sampling method may have introduced sampling bias, thereby limiting the representativeness of the sample and the generalizability of the findings. Second, the study only included nursing students as the study population; this single-group selection limits the applicability of the findings to students from other academic disciplines. Third, the sample was drawn from four medical universities in Northeast China, which may further restrict the applicability of the findings to other regions of China or different cultural contexts, such as southern provinces or areas with greater cultural variation. Fourth, as with all self-reported questionnaire studies, the data may be subject to social desirability bias.

From a methodological perspective, this study did not conduct measurement invariance testing across groups; therefore, the structural equivalence of the scale across different grades or disciplines (e.g., age, gender, etc.) cannot be fully confirmed. In addition, the cultural adaptation process in this study relied primarily on quantitative methods; incorporating qualitative approaches may help to further explore the semantic meaning and contextual adaptability of the items in different cultural settings. To more comprehensively evaluate the reliability and validity of the scale, future studies should consider multicenter research involving different regions, cultural backgrounds, and multidisciplinary populations to further verify its generalizability, and employ longitudinal designs to examine its measurement stability.

## 5 Conclusion

This study strictly followed the Brislin translation model and successfully introduced the STISS scale, which demonstrated strong reliability and validity in the Chinese cultural context. The C-STISS can serve as an effective and reliable tool for nursing educators and managers to assess nursing students’ level of stigmatization toward individuals with sexually transmitted infections (STIs). This tool not only helps nursing educators and managers understand nursing students’ stigmatization tendencies when facing STI patients but also provides valuable insights for optimizing nursing education and training. Based on the measurement results of this scale, nursing educators and managers can further improve training strategies and teaching methods, and develop intervention measures (such as role-playing, demonstrations, and empathy skill development) to foster a non-discriminatory and equal attitude among nursing students toward individuals with STIs, ultimately improving the quality of nursing services.

## Data Availability

The datasets presented in this article are not readily available because this dataset is not publicly available due to policy and privacy restrictions. Requests to access the datasets should be directed to panyue19900125@163.com.
