# Supplementary material for: The cross-cultural adaptation and psychometric evaluation of the Chinese version of the stigma scale for caring for individuals with sexually transmitted infections (STISS)—student version: a translation and validation study
Source: Front Psychol. 2026 Jul 21;17:1858646. doi: 10.3389/fpsyg.2026.1858646 (PMC13433436; doi:10.3389/fpsyg.2026.1858646)
Supplement: Supplementary file 1 [file Table_1.DOCX]

Table 1. Skewness and Kurtosis of Each Item on the Chinese Version of the Stigma Scale for Caring for Individuals with Sexually Transmitted Infections (C-STISS)

| **Item** | Skewness | Kurtosis |
| --- | --- | --- |
| B1 | -0.813 | 0.303 |
| B2 | -0.943 | 0.480 |
| B3 | -0.828 | 0.097 |
| B4 | -0.812 | 0.179 |
| B5 | -0.840 | 0.074 |
| B6 | -0.790 | 0.168 |
| B7 | -0.955 | 0.437 |
| B8 | -0.940 | 0.364 |
| B9 | -0.890 | 0.514 |
| C1 | -0.773 | -0.138 |
| C2 | -0.720 | -0.497 |
| C3 | -0.742 | -0.267 |
| PR1 | -0.677 | -0.173 |
| PR2 | -0.674 | -0.213 |
| PR3 | -0.557 | -0.488 |
| PR4 | -0.509 | -0.438 |
| PR5 | -0.522 | -0.511 |
| SS1 | -0.864 | 0.014 |
| SS2 | -0.732 | -0.272 |
| SS3 | -0.710 | -0.355 |

Note: B: Blame; C: Concealment; PR: Personal Response; SS: Segregation from Society.

Table 2. Item-Total Statistics of the Chinese Version of the Stigma Scale for Caring for Individuals with Sexually Transmitted Infections (C-STISS)

| Item | CITC | Cronbach’s α if item deleted |
| --- | --- | --- |
| B1 | 0.607 | 0.917 |
| B2 | 0.566 | 0.918 |
| B3 | 0.630 | 0.916 |
| B4 | 0.667 | 0.915 |
| B5 | 0.625 | 0.916 |
| B6 | 0.605 | 0.917 |
| B7 | 0.590 | 0.917 |
| B8 | 0.547 | 0.918 |
| B9 | 0.613 | 0.917 |
| C1 | 0.582 | 0.917 |
| C2 | 0.553 | 0.918 |
| C3 | 0.531 | 0.918 |
| PR1 | 0.579 | 0.917 |
| PR2 | 0.543 | 0.918 |
| PR3 | 0.594 | 0.917 |
| PR4 | 0.594 | 0.917 |
| PR5 | 0.572 | 0.917 |
| SS1 | 0.554 | 0.918 |
| SS2 | 0.540 | 0.918 |
| SS3 | 0.564 | 0.918 |

**Note**: The overall Cronbach's α coefficient for the scale was 0.921.

**C-STISS**

|  | | Strongly disagree | Disagree | Neutral | Agree | Strongly agree |
| --- | --- | --- | --- | --- | --- | --- |
| **Dimension 1: Blame** | | | | | | |
| Item1 | Compared to caring for patients with sexually transmitted infections, I feel more confident in caring for other patients |  |  |  |  |  |
| Item2 | I may unwillingly exhibit averted body language when I have to care for an individual with STIs |  |  |  |  |  |
| Item3 | Despite being a healthcare professional, I may react aversively towards individuals with STIs |  |  |  |  |  |
| Item4 | When caring for individuals with STIs, I try to avoid communicating with them |  |  |  |  |  |
| Item5 | I find it difficult to identify with individuals with STI |  |  |  |  |  |
| Item6 | I find it difficult to show compassion to a person with STI |  |  |  |  |  |
| Item7 | When I learn that my close friend has been diagnosed with STI, I try to stay away from him/her |  |  |  |  |  |
| Item8 | When I learn that someone in my family has been diagnosed with STI, I try to stay away from them |  |  |  |  |  |
| Item9 | I think these people got an STI because of their lifestyle |  |  |  |  |  |
| **Dimension 2: Concealment** | | | | | | |
| Item10 | If I am diagnosed with an STI, I will not tell any of my colleagues |  |  |  |  |  |
| Item11 | If I am diagnosed with an STI, I might be more willing to seek help from healthcare professionals who are not affiliated with my own institution |  |  |  |  |  |
| Item12 | If I get an STI, I tell my relatives to keep it a secret |  |  |  |  |  |
| **Dimension 3: Personal Response** | | | | | | |
| Item13 | I feel uncomfortable working with a friend who has an STI |  |  |  |  |  |
| Item14 | If I have an STI and I am unable to cope with it, I will view it as a burden |  |  |  |  |  |
| Item15 | If I am diagnosed with an STI, I would consider it an unfortunate event |  |  |  |  |  |
| Item16 | If I get an STI, I think it is a punishment for me |  |  |  |  |  |
| Item17 | If I get an STI, I will distance myself from those around me |  |  |  |  |  |
| **Dimension 4: Segregation from Society** | | | | | | |
| Item18 | I think individuals with STIs may be a burden to society |  |  |  |  |  |
| Item19 | When a person with an STI is receiving treatment, I would avoid having them share a room with other patients |  |  |  |  |  |
| Item20 | When I care for individuals with STIs, I take additional precautions |  |  |  |  |  |
